# Preparation of Liposomes from Soy Lecithin Using Liquefied Dimethyl Ether

**DOI:** 10.3390/foods10081789

**Published:** 2021-08-02

**Authors:** Hideki Kanda, Tsubasa Katsube, Wahyudiono, Motonobu Goto

**Affiliations:** Department of Materials Process Engineering, Nagoya University, Furocho, Chikusa, Nagoya 464-8603, Japan; mdzyw18342@yahoo.co.jp (T.K.); wahyudiono@b.mbox.nagoya-u.ac.jp (W.); goto.motonobu@material.nagoya-u.ac.jp (M.G.)

**Keywords:** subcritical fluid, vesicle, phospholipid, lipid bilayer

## Abstract

We investigated a method to prepare liposomes; soy lecithin was dissolved in liquefied dimethyl ether (DME) at 0.56 MPa, which was then injected into warm water. Liposomes can be successfully prepared at warm water temperatures above 45 °C. The transmission electron microscopy (TEM) images of the obtained liposomes, size distribution, ζ-potential measurements by dynamic light scattering and the amount of residual medium were compared by gas chromatography using the conventional medium, diethyl ether. The size of the obtained liposomes was approximately 60–300 nm and the ζ-potential was approximately −57 mV, which was almost the same as that of the conventional medium. Additionally, for the conventional media, a large amount remained in the liposome dispersion even after removal by depressurization and dialysis membrane treatment; however, liquefied DME, owing to its considerably low boiling point, was completely removed by depressurization. Liquefied DME is a very attractive medium for the preparation of liposomes because it does not have the toxicity and residue problems of conventional solvents or the hazards of ethanol addition and high pressure of supercritical carbon dioxide; it is also environmentally friendly.

## 1. Introduction

Liposomes are spherical vesicles with one or more phospholipid bilayers that surround an aqueous compartment. Liposomes can encapsulate aqueous hydrophilic or hydrophobic substances within the phospholipid bilayer and are often classified as small unilamellar vesicles (SUVs), large unilamellar vesicles (LUVs), or multilamellar vesicles (MLVs), based on their size and the number of phospholipid bilayers [1]. SUVs and LUVs have diameters in the range of 20–100 and 100–1000 nm, respectively [2], and are often used as biomaterial carriers [3]. Liposomes are also tools for the oral delivery of nutraceuticals because of their unique properties. Ordinary tablets and capsules have low absorption and bioavailability properties; thus, the encapsulating fat-soluble and water-soluble nutrients in liposomes are investigated for the ability to prevent their destruction in the digestive system and facilitate their delivery to cells and tissues. Liposomes can contain a wide variety of hydrophilic and hydrophobic diagnostic and therapeutic agents, providing a relatively large drug payload per particle and protecting the encapsulated drug from metabolic processes [4,5,6].

The conventional methods of preparing liposomes require toxic organic solvents, complex multistage processes and high energy costs and the resulting liposomes usually show poor stability. In previous studies, ethanol and diethyl ether were used as organic solvent injection methods. In the ethanol injection method, phospholipids were dissolved in ethanol and rapidly injected into water to produce liposomes. Liposomes with diameters ranging from 30 to 110 nm were obtained using this method. One problem is that ethanol mixes azeotropically with water, making it difficult to completely remove ethanol from the liposome dispersion [7,8]. Moreover, the removal of ethanol from an aqueous liposome solution requires the distillation of ethanol and water by heating; this results in the destruction of liposomes and encapsulated biomaterials. Additionally, ethanol is not suitable in various religions and carries risks that prevent its use. In the ether injection method, phospholipids were dissolved in diethyl ether and the mixture was injected into water. When the water was heated to 55–65 °C, the diethyl ether evaporated and liposomes were formed. The diameter of the liposomes prepared by this method was approximately 70–190 nm [9]. The diethyl ether injection method has been used to encapsulate 5.6-carboxyfluorescein, a hydrophilic drug [10] and a plasmid, a part of the DNA molecule [11]. However, there is concern that the ether may remain in the encapsulated biomaterials. Additionally, diethyl ether must be heated to a considerably high temperature to remove the solvent, making it unsuitable for the encapsulation of heat-sensitive materials. Furthermore, diethyl ether is considerably difficult to handle because it easily produces explosive peroxides and is toxic [1,2]. To solve these problems, the use of organic solvents with low boiling points, such as methyl ethyl ether (boiling point, 10.8 °C) and dichlorofluoromethane (boiling point, 8.9 °C), has been considered [8]. However, methyl ethyl ether has the same peroxide problem as diethyl ether and dichlorofluoromethane has been banned because of its ozone depletion and greenhouse effect, which is thousands of times stronger than that of carbon dioxide. 

To solve this problem, the use of supercritical carbon dioxide (SC-CO_2_) as a green solvent instead of organic solvents has been proposed. CO_2_ is not only easy to separate from the product but has also a low critical temperature, providing a mild operating temperature suitable to process and encapsulate thermophilic compounds. Previous studies have shown that various nutraceutical ingredients have been encapsulated in liposomes using SC-CO_2_ [12,13,14,15,16,17]. However, because SC-CO_2_ is non-polar, the solubility of polar phospholipids and water is considerably low; therefore, to incorporate phospholipids and water into SC-CO_2_, ethanol is mixed with the SC-CO_2_. For example, several researchers used the supercritical reversed-phase evaporation method to prepare liposome aqueous dispersions by emulsification, by adding water to a homogeneous mixture of SC-CO_2_/phospholipid/ethanol with sufficient agitation, followed by decompression [18,19,20,21]. However, because ethanol is required when SC-CO_2_ is used as an alternative solvent, the problem of ethanol contamination of aqueous liposome solution arises again. In recent years, a method to prepare liposomes using SC-CO_2_ as a solvent, without using ethanol, by introducing ultrasonication has been investigated [22]. However, the disadvantage of SC-CO_2_ with ultrasonication is the risk of an accident when the bolts of the pressure vessel loosen owing to vibrations caused by ultrasonic irradiation. This is because the operating pressure is 10–20 MPa and leakage from inside the vessel must be avoided.

In other words, the essence of the problem is the following. Organic solvents should be heated for evaporation; however, they have hazardous environmental impacts. SC-CO_2_ is nonpolar and its operating pressure is significantly high. Therefore, we thought that one solution would be to create liposomes using a slightly polar substance that has a significantly low boiling point, is a gas at room temperature, is easily volatile and is not toxic to living organisms. To satisfy these conditions, we focused on dimethyl ether (DME) as a candidate substance.

Notably, DME is different from diethyl ether, which is commonly called ether, and its properties are also significantly different. DME is the simplest form of ether and has characteristics such as a low boiling point (–24.8 °C) [23]. Therefore, when DME is used as the extraction solvent, it is liquefied [24,25,26,27,28,29,30]. At room temperature, DME does not remain in the extracted materials [29,30]. The absence of the biological toxicity of DME has been confirmed by the bioassay evaluation of DME-dissolved water using a microbial culture [30]. DME is a safe extraction solvent for food production and has residue standards for food [31]. Furthermore, DME has excellent properties, such as zero ozone depletion potential and low global warming potential [32]. Therefore, DME has attracted attention as a new green solvent [33]. Additionally, DME has a bent molecular structure; therefore, it has weak polarity. For example, DME is known to form weak hydrogen bonds. DME dimers have small triply C−H···O−C improper bonds [34]. When water and liquefied DME come into contact, the oxygen of DME is the hydrogen-bond acceptor and the hydrogen of water is the hydrogen-bond donor. Therefore, liquefied DME can partially mix with water and its solubility in water is significantly low in its gaseous state [35,36]. Additionally, DME can extract polar substances from wet microalgae [37]. This indicates that phospholipids may also interact with liquefied DME. However, in recent years, attempts have been made to use this liquefied DME for purposes other than that of extraction medium; for example, it has been used as an antisolvent in amino acid crystallization [38], or a draw solute in forward osmosis [39]. To the best of our knowledge, no attempt has been made to apply liquefied DME to the preparation of liposomes.

In this study, for the first time, we attempted to prepare liposomes by applying liquefied DME as a new medium; this is a method to avoid the problems of the high pressure of SC-CO_2_ and ethanol mixing in the liposome preparation. The expected operating pressure is approximately 0.5–0.6 MPa, which is much milder than that of SC-CO_2_ and there is no danger of the loosening of the pressure vessel because of ultrasound irradiation. The preparation method for liposomes is based on the simplest injection method. The properties of the DME-prepared liposomes were compared with those of the conventional method using diethyl ether with the same equipment. The properties and storage stability of the obtained liposomes were verified and the amount of residual media was verified.

## 2. Materials and Methods

### 2.1. Samples and Chemicals

A mixture of soy lecithin (Wako Pure Chemical Industries, Ltd., Osaka, Japan) and cholesterol (90%; Wako Pure Chemical Industries, Ltd.) was used as the raw material for liposomes. Soy lecithin is a group of phospholipids mainly consisting of phosphatidylcholine (PC), phosphatidylethanolamine (PE) and phosphatidic acid (PA) purified from soybeans. According to the manufacturer, the components were 38% of PC, 30% of PE, 25% of PA and 7% of other phospholipids. These are commonly used as raw materials for liposomes [40]. The phase transition temperature of the soybean lecithin was measured using a differential scanning calorimeter (DSC-60, Shimadzu Co., Kyoto, Japan) in a nitrogen atmosphere at a heating rate of 3 °C/min from 0 °C to 70 °C and it was 27.7 °C.

Liquefied DME (supplied as a propellant filled in a spray-work air can 420D) was purchased from Tamiya, Inc., Shizuoka, Japan, and was used without further purification. Diethyl ether (Wako Pure Chemical Industries, Ltd.), the DME comparator, had a purity of 99.5%.

### 2.2. DME Injection Method

**Figure 1:**
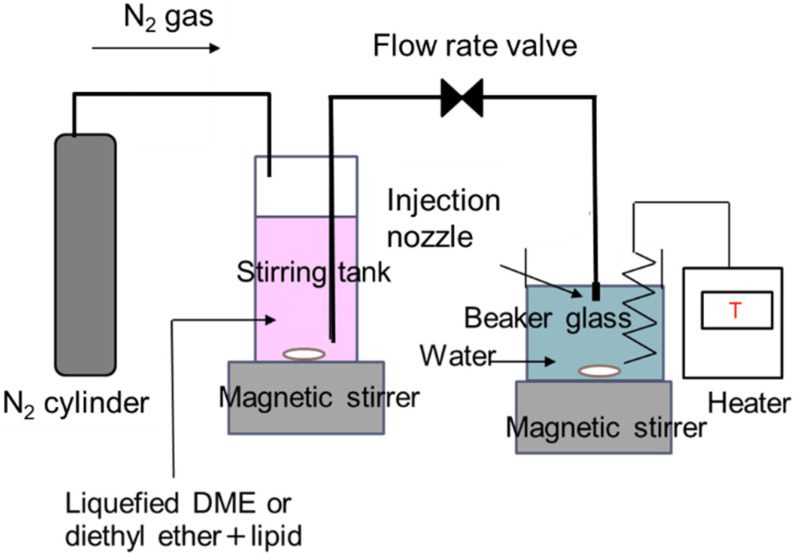
Schematic diagram of the liposome preparation system using liquefied DME.

A schematic diagram of the experimental setup for liposome preparation by the solvent injection method using liquefied DME as a medium is shown in Figure 1. The apparatus consisted of a nitrogen cylinder, a stirring tank (96 mL, HPG96-3, Taiatsu Techno Corp., Saitama, Japan), 300 g of purified water in a glass beaker and an injection nozzle installed in the purified water. These were connected in series using 1/16-inch SUS tubes. The stirring tank consisted of a transparent, pressure-resistant glass container coated with polycarbonate. The stirring tank was filled with 60 mg of a mixture of soy lecithin and cholesterol in a 4:1 weight ratio and 40 ± 0.5 g of the liquefied DME or diethyl ether and it was stirred at 400 rpm and 23 °C for 20 min to ensure that the lipid mixture was completely dissolved in the medium. The internal pressure in the stirring tank was 0.56 MPa [23], which is the saturated vapor pressure for liquefied DME. An injection nozzle (2.5 mm in diameter) was placed 1.5 cm below the water surface in the glass beaker. The water temperature in the glass beaker was maintained at 60 ± 2 °C using a temperature controller and heater (300 W, CUNH-1103, Izumi Dennetsu Co., Ltd., Osaka, Japan). To clarify the effect of evaporation temperature, the slimmer test was conducted at 30 °C and 45 °C for comparison. Lipid-dissolved medium was injected into the water by pushing the surface of the medium in the stirred tank to the injection nozzle using nitrogen gas at 0.7 MPa. The injection speed was 10 mL/min and was adjusted using a manual flow rate valve by observing the decreasing rate of the medium level inside the stirred tank, using the volume memory printed on the transparent wall of the stirred tank. The injected medium was evaporated in water. The liposome dispersion was filtered using an aspirator (AS-01, AS ONE Corp., Osaka, Japan) and filter paper (0.4 μm pore size, Kiriyama filter paper, No. 5B, Kiriyama Glass Works Co., Tokyo, Japan) under reduced pressure to remove excess lipids. Afterward, the liposome dispersion was decompressed in a rotary evaporator (N-1200A, EYELA, Tokyo, Japan) and a warm bath (SB-1200, EYELA) for 30 min to completely evaporate the medium. The obtained liposome dispersion was dialyzed for 3 days using a dialysis membrane (fractional molecular weight = 3500; Spectra/Por^®^ 3, Repligen Japan, Ritto, Japan) to remove residual media from the aqueous phase outside the liposomes. The series of steps from liposome preparation to analysis was repeated three times to check their reproducibility. 

### 2.3. Analysis

#### 2.3.1. Transmission Electron Microscopy (TEM) Observation

The obtained liposome dispersions were dried on a Cu microgrid for TEM observation (NP-C15, Okenshoji Co., Ltd., Tokyo, Japan) and observed using a TEM (JEM2100H/FK, JEOL, Ltd., Tokyo, Japan) at an acceleration voltage of 200 kV. When the liposome dispersion was dried on the TEM microgrid, the liposomes were stained using the negative staining method described below [41]. In the negative staining method, the TEM microgrids were hydrophilized by discharging 100 V AC at 5 A for 10 s using an electron microscope hydrophilization system (DII-29020HD, JEOL, Ltd., Tokyo, Japan). A Parafilm^®^ was floated on the water at 80 °C in a water bath and 0.5 mL of the liposome dispersion, purified water and 2 wt% phosphotungstic acid solution (Nacalai Tesque, Inc., Kyoto, Japan) were dropped onto the Parafilm^®^. The hydrophilized TEM microgrid was contacted with a droplet of liposome dispersion heated to 80 °C for 5 s. Subsequently, excess droplets adhering to the TEM grid were removed by absorbing the water with a filter paper and the TEM grid was dried. Thereafter, a similar procedure was repeated with droplets of purified water and 2 wt% phosphotungstic acid solution. Finally, the TEM microgrids were dried in a desiccator for 12 h.

#### 2.3.2. Dynamic Light Scattering (DLS) for Size Distribution and ζ-Potential Analysis

The size distribution and ζ-potential of liposomes were measured at 25 °C using a DLS particle size analyzer (Zetasizer Nano-ZS, Malvern Instruments Ltd., Worcestershire, UK). The refractive indices of liposomes and pure water were set to 1.45 and 1.33 for analysis, respectively [42,43].

#### 2.3.3. Residual Medium Analysis

The liposome dispersion (1 mL) was placed in a gas chromatography (GC) headspace vial and 1 mL of a surfactant, Tween^®^20 (polyoxyethylene (20) sorbitan monolaurate) solution (10 wt%, Tokyo Chemical Industry Co., Ltd., Tokyo, Japan) was added. Afterward, the mixture was vigorously shaken and allowed to stand for 10 min to disrupt the liposomes [44]. Thus, the residual medium inside the liposome was released. The released medium was evaporated by holding at 55 °C for 30 min in a vial. Using a microsyringe heated to 60 °C, 100 μL of the gas phase was obtained from the vial and analyzed by GC. 

Following a previous study [45,46], the amount of residual media was analyzed using GC coupled with a flame ionization detector (GC-2014, Shimadzu Co., Kyoto, Japan). A methyl polysiloxane capillary column (HP-5MS; 30 m × 250 µm (internal diameter) × 0.25 µm, Agilent Technologies Tokyo Ltd., Tokyo, Japan) was employed. The oven temperature was initially set at 100 °C for 45 min and increased to 160 °C at a rate of 10 °C/min. The inlet temperature was 200 °C and the detection temperature was 160 °C. 

The amount of residual media was also measured when the dialysis membrane was not used, because the removal of excess media from the liposome by the dialysis membrane was time-consuming. In this case, the media was removed only by decompression using a rotary evaporator and the decompression time was changed to 0, 10 and 20 min to measure the change over time. The pretreatment conditions using Tween20^®^ and the detection conditions using GC were the same.

## 3. Results

**Figure 2:**
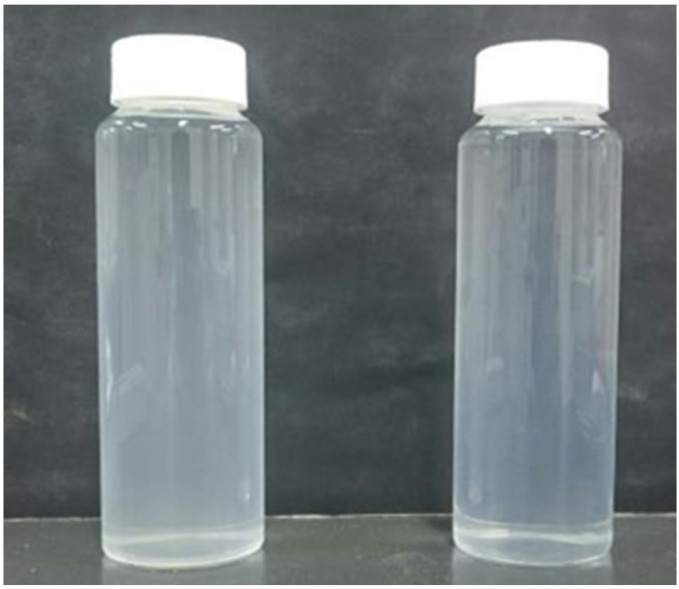
Liposome dispersions prepared using liquefied DME (**left**) and diethyl ether (**right**).

The liposome dispersions prepared using liquefied DME and diethyl ether are shown in Figure 2. The prepared liposome solution had a white transparent dispersion in both methods. The following analyses were performed on these aqueous liposome solutions.

### 3.1. TEM Observation 

**Figure 3:**
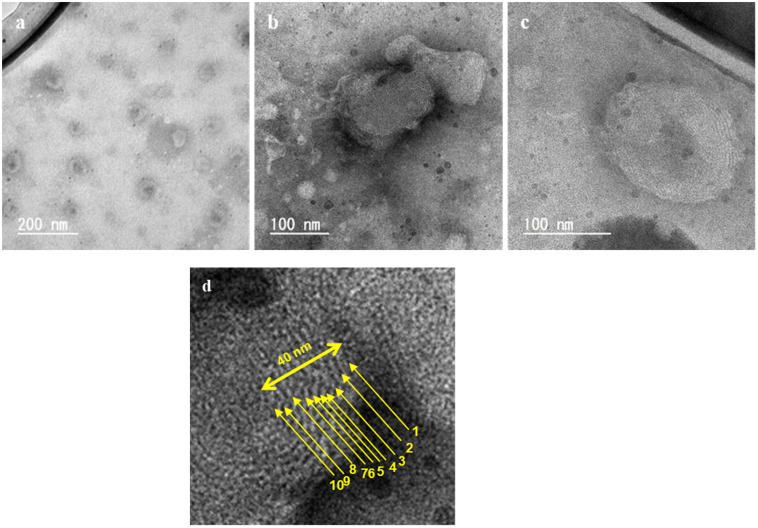
TEM images of the liposomes prepared by the liquefied DME method at 60 °C. Magnification (**a**) × 100 k, (**b**) × 200 k and (**c**) × 300 k. (**d**) Enlarged view of (**b**), with emphasis on clarity and contrast.

Figure 3 shows the TEM images of the liposomes prepared using liquefied DME at 60 °C, which is the typical image observed by negative staining [41]. The boundary between the liposome and its surroundings, the structure of the lipid membrane and the portion of the liposome interior caused by the aqueous phase were observed. The black contrast in the central part was due to the scattering of electron beams by the phosphotungstic acid that penetrated the liposome during staining, indicating that the aqueous phase existed before drying [41]. As shown in Figure 3a, we observed liposomes with a diameter of approximately 60 nm and a trace of an aqueous phase in the center. Figure 3b shows liposomes with a diameter of approximately 150 nm and a dense lipid membrane, with approximately 10 layers with a thickness of 4 nm, as shown in Figure 3d. Figure 3c shows liposomes with a diameter of approximately 170 nm, a trace of an aqueous phase in the center. Since the typical thickness of lipid membrane is 4–6 nm, this layer can be regarded as the lipid membrane of liposomes. 

**Figure 4:**
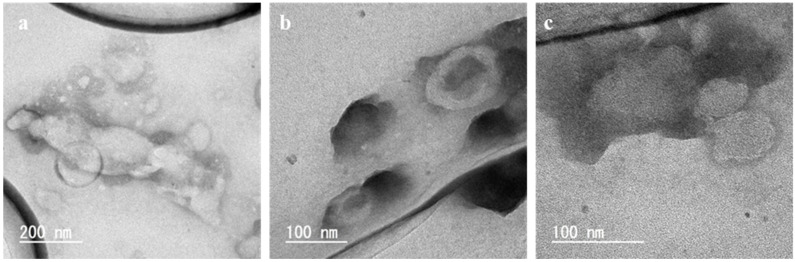
TEM images of the liposomes prepared by the conventional diethyl ether method at 60 °C. Magnification (**a**) × 100 k, (**b**) × 200 k and (**c**) × 300 k.

Figure 4 shows the TEM images of the liposomes prepared using diethyl ether at 60 °C. In Figure 4a, a LUV with a single lipid membrane was observed. In Figure 4b, MLVs with traces of aqueous phase in the center were observed. In Figure 4c, liposomes with lipid membranes ranging from 50 to 125 nm were observed and the thickness of the lipid membrane was approximately 6 nm. The TEM images of these liposomes are not so different from those of the liposomes prepared using liquefied DME (Figure 3).

**Figure 5:**
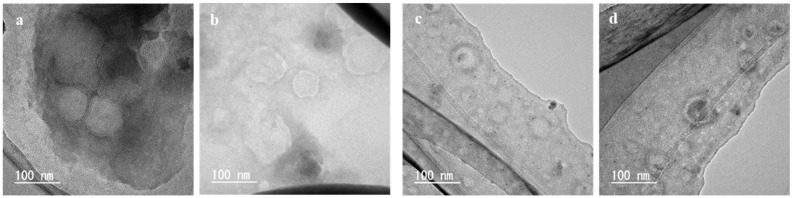
TEM images of the liposomes prepared by (**a**,**b**) the liquefied DME and (**c**,**d**) diethyl ether method at 45 °C. Magnification × 200 k.

Figure 5 shows the TEM images of the liposomes prepared using liquefied DME and diethyl ether at 45 °C. For the liquefied DME (Figure 5a,b), liposomes of approximately 100 nm and 2–3 lipid layers with a thickness of approximately 5 nm were observed. For diethyl ether (Figure 5c,d), liposomes with lipid films in the range of 50–100 nm were observed. Liposomes with traces of the aqueous phase were observed for the liquefied DME and diethyl ether. The TEM images of these liposomes were not very different from those of the liposomes prepared at 60 °C (Figure 3 and Figure 4).

**Figure 6:**
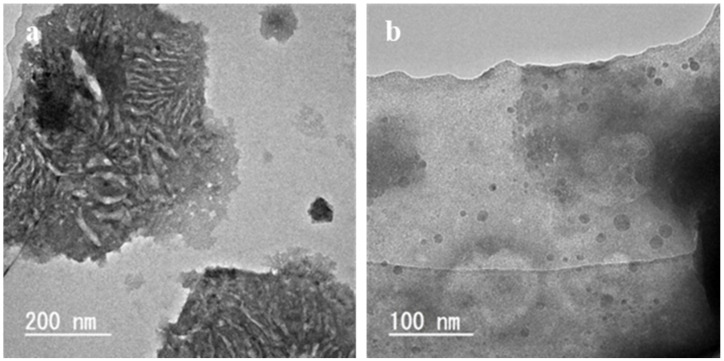
TEM images of the liposomes prepared by the liquefied DME method at 30 °C. Magnification (**a**) × 100 k and (**b**) × 200 k.

Figure 6 shows the TEM images of the liposomes prepared using liquefied DME at 30 °C. Notably, the boiling point of diethyl ether is 34.8 °C; therefore, liposomes could not be created at 30 °C, thus TEM observation was not performed. When prepared with liquefied DME at 30 °C, lipid aggregates (Figure 6a) and liposomes (Figure 6b) were observed. The aggregates shown in Figure 6a had a diameter of 600–700 nm and rod-like structures with a length of 30–100 nm were observed inside the aggregates. In Figure 6b, in addition to those with a diameter of approximately 100 nm, similar to those observed in the preparations at 60 °C and 45 °C, several small spherical materials with a diameter of approximately 30–50 nm, which may not be liposomes, were observed.

### 3.2. DLS for Size Distribution and ζ-Potential Analysis

**Figure 7:**
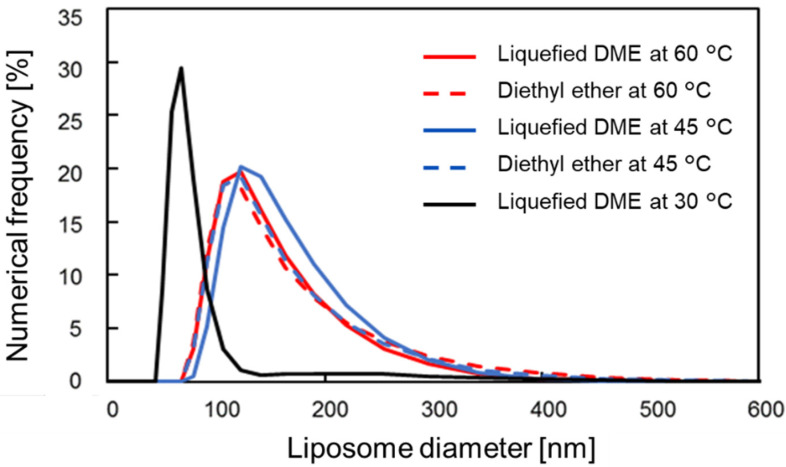
Diameter distribution of the liposome prepared using liquefied DME and diethyl ether.

Figure 7 shows the DLS size distributions of the liposomes obtained using liquefied DME and diethyl ether. There was no significant difference in the size distributions of the liquefied DME and diethyl ether liposomes and the distribution of the liposomes ranged from 60 to 350 nm. At 60 °C, the average liposome sizes were 153 nm for liquefied DME and 162 nm for dimethyl ether, indicating no significant difference. The polydispersity index (PDI) of these liposomes was 0.12 ± 0.01 for both liquefied DME and dimethyl ether. These values are smaller than the PDI after conversion of heterogeneous liposomes to homogeneous liposomes in the microchannel laminar flow with sonication [47], indicating that very homogeneous liposomes were prepared by injection method using liquefied DME. The diameters of the liposomes obtained by DLS were generally consistent with those from the TEM observations, confirming the validity of the results.

Conversely, when the liposomes were prepared using liquefied DME at 30 °C, the size distribution by DLS showed a medium liposome size of 78 nm and a large number of smaller materials were produced. It was also implied that there was a small amount of material in the 100–300 nm range. These results were also consistent with a large number of small clumps and large aggregates observed in the TEM images.

The ζ-potentials of the liposome prepared using liquefied DME and diethyl ether were −57.2 and −57.9 mV and no significant difference was observed. In general, particles are stable above 30 mV or below −30 mV in their dispersion [48]. Therefore, these DME liposomes had a negative potential and were dispersed with high repulsion among the liposomes. In other words, the size and dispersive stability of the liposomes obtained using liquefied DME were comparable to those obtained using the conventional technique, suggesting that liposomes can be successfully prepared. 

The ζ-potentials of the liposomes prepared using liquefied DME and diethyl ether at 45 °C were −62.4 and −60.5 mV and that of the liposomes prepared using liquefied DME at 30 °C was −58.8 mV. 

### 3.3. Residual Medium Analysis

After the final treatment with the dialysis membrane, the residual medium could not be detected by GC in the liposomes prepared using liquefied DME. This means that the residual solvent was less than 0.2 mg/L. Conversely, in the liposomes prepared using diethyl ether, there was 0.683 mg/L of residual solvent in the liposomes.

**Figure 8:**
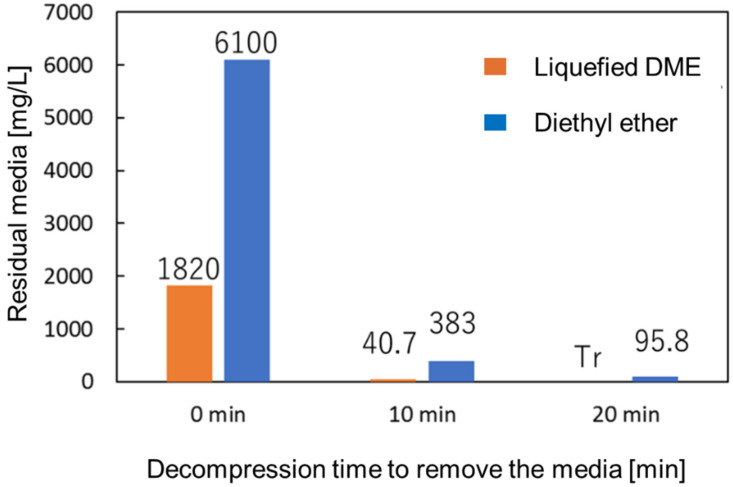
Residual media in aqueous liposome solution for only decompression treatment without dialysis membrane.

Without the final treatment using the dialysis membrane, before the decompression treatment using the rotary evaporator immediately after liposome preparation, the residual media in the entire aqueous liposome solution prepared using liquefied DME was 1820 mg/L and that using diethyl ether was 6100 mg/L, as shown in Figure 8. After 10 min of decompression, the residual DME rapidly decreased to 40.7 mg/L. Similarly, the residual diethyl ether concentration was 383 mg/L. After 20 min of decompression, the residual DME decreased to a relatively low limit of detection (<0.2 mg/L). In contrast, the residual diethyl ether was detected to be 95.8 mg/L.

### 3.4. Stability in a Large Storage Period

**Figure 9:**
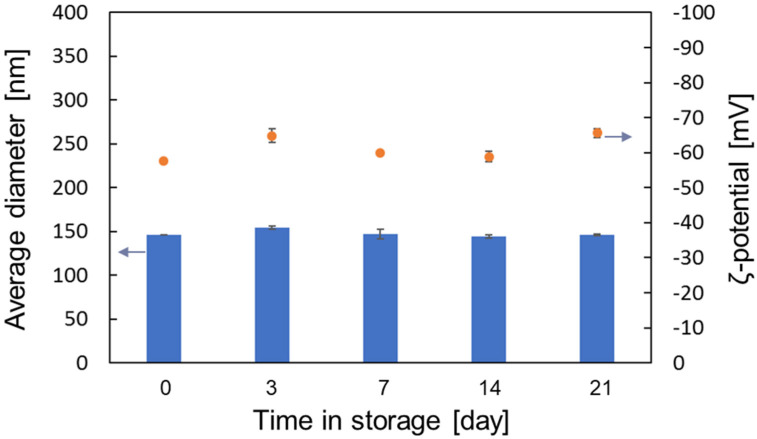
Medium diameter and ζ-potential of liposomes prepared with liquefied DME and stored at 4 °C.

**Figure 10:**
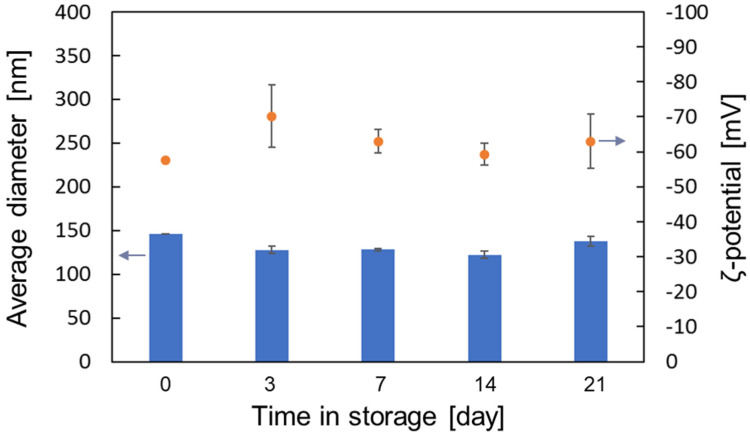
Medium diameter and ζ-potential of liposomes prepared with liquefied DME and stored at 37 °C.

In order to verify the long-term storage stability of liposomes prepared with liquefied DME, they were stored at 4 °C and 37 °C for up to 21 days and the medium diameters and ζ-potentials were measured by DLS at various storage times. The results on the stability of liposomes stored at 4 °C and 37 °C are shown in Figure 9 and Figure 10, respectively. As shown in Figure 9 and Figure 10, the average diameter and ζ-potential of liposomes did not change significantly when stored at 4 °C or 37 °C, indicating that liposomes prepared using liquefied DME as a medium were stably dispersed for a long time.

## 4. Discussion

In this study, liposomes were prepared using liquefied DME instead of conventional organic solvents, which are toxic and difficult to handle and they cause a large environmental impact and religious problems [7,8,9], and SC-CO_2_, which requires the addition of ethanol [18,19,20,21], high operating pressure and sonication to dissolve phospholipids [22]. First, the TEM observation and DLS measurements of size and ζ-potential showed that it was possible to prepare liposomes with almost the same properties as those of conventional diethyl ether, even when using liquefied DME. The pressure in the vessel of the liquefied DME is 0.56 MPa, which is considerably lower than the operating pressure of SC-CO_2_ (10–20 MPa) [13,14,15,16,17,18,19,20,21,22]. The high safety of DME can be seen from the fact that the container used in this test was made of glass. 

The temperature of the pure water into which the medium is injected should be 45 °C or higher. It cannot be denied that liposomes can be created at water temperatures between 30 and 45 °C. Conversely, at 30 °C, which is well above the boiling point of liquefied DME, the aggregates of lipids other than liposomes were observed by TEM because of the phase transition temperature of phospholipids. Phospholipid bilayers become more flexible, fluid and permeable at temperatures above the phase transition temperature. Therefore, it is preferable to prepare liposomes at or above the phase-transition temperature [49]. The phase transition temperature of the soybean lecithin used in this experiment was 27.7 °C, as mentioned in Section 2.1, which is close to the injection temperature of 30 ± 2 °C. Although the pure water was accurately controlled, the heat of vaporization may cause a local decrease in temperature near the injection nozzle, where the liquefied DME vaporizes. This local temperature decrease may have inhibited liposome formation because the lipid membrane was not sufficiently flexible.

The amount of residual medium was measured by GC and, for diethyl ether, the residual medium was observed even after the medium was removed using decompression and dialysis membrane treatment. This suggests that the medium remained inside the liposome, which is a problem in terms of biotoxicity and transporting functional materials into the body. In contrast, it was suggested that liquefied DME can completely remove the residual medium from the inside of the liposome without using a dialysis membrane, just by a 20-minute decompression operation. In addition to the fact that DME-dissolved water has considerably low biotoxicity [30], the result showing that the residual amount was below the detection limit indicates that the method of preparing liposomes using liquefied DME is superior to that using conventional organic solvents in terms of biocompatibility.

Additionally, previous studies have shown that liquefied DME can extract a variety of functional substances, such as astaxanthin [50,51], fucoxanthin [52], lutein [29,53], resveratrol and its glycosides [54], catechins and caffeine [55], tocopherol [56], gingerols and capsaicin [27]. Among these substances, carotenoids, such as astaxanthin, fucoxanthin and lutein, are hydrophobic, therefore difficult to encapsulate into liposomes by mechanical methods. Therefore, a medium to dissolve them is necessary to encapsulate them into liposomes. Moreover, the solubility of these carotenoids in SC-CO_2_ is so extremely low that SC-CO_2_ is used as an anti-solvent for crystallizing carotenoids [57,58,59]. In the future, these functional substances and natural pigments that are difficult to handle with SC-CO_2_ are expected to be dissolved in liquid DME together with soy lecithin and cholesterol and injected into warm water to be encapsulated in liposomes.

## 5. Conclusions

In this study, liposomes were prepared by dissolving soy lecithin and cholesterol in liquefied DME and injecting them into warm water. As a result of the TEM image observation, size distribution measurement by DLS and ζ-potential measurement, it was found that the liquefied DME injection method succeeded in producing liposomes similar to those produced using conventional diethyl ether at above 45 °C. We also measured the amount of residual DME and found that DME was no longer detected after only 20 min of the depressurization of the liposome dispersion. In contrast, with conventional diethyl ether, a large amount of diethyl ether remained even after depressurization and dialysis membrane treatment. Considering the considerably low boiling point of DME and the low toxicity of DME to living organisms, even if it remains, the safety of the liquefied DME injection method is much higher than that of the conventional method. Furthermore, the operating pressure of the liquefied DME injection method is much lower than that of SC-CO_2_, which is also known as a green medium, and ethanol is not required. In other words, in comparison with SC-CO_2_, DME is a superior medium in terms of ethanol contamination and safety during operation.

## Data Availability

Not applicable.
